# A longitudinal analysis of the impact of nonsurgical and surgical treatment of peri‐implantitis upon clinical parameters and implant stability quotient values. A 2–3‐year follow‐up

**DOI:** 10.1002/cre2.833

**Published:** 2024-02-03

**Authors:** Peter Harrison, Edward Madeley, Michael Nolan, Stefan Renvert, Ioannis Polyzois

**Affiliations:** ^1^ Division of Restorative Dentistry & Periodontology, Dublin Dental University Hospital Trinity College Dublin Dublin Ireland; ^2^ Department of Health Sciences Kristianstad University Kristianstad Sweden; ^3^ Blekinge Institute of Technology Karlskrona Sweden

**Keywords:** dental implants, L‐PRF, peri‐implantitis, surgical treatment

## Abstract

**Objectives:**

In this study, the aim was to investigate the medium‐ to long‐term impact of peri‐implantitis treatment upon clinical parameters and implant stability quotient values and to ascertain if magnetic resonance frequency analysis can be used as a diagnostic tool to demonstrate postoperative healing following treatment of peri‐implantitis.

**Materials and Methods:**

A total of *n* = 26 patients (*n* = 86 implants) diagnosed with peri‐implantitis were recruited for this prospective cohort study and four different treatment modalities were used. Baseline measurements of a number of clinical parameters as well as implant stability measurements in the form of ISQ were recorded. These measurements were repeated at 6, 12, and 24–36 months following treatment. Analysis of variance was performed for all implants treated as well as separately for each treatment modality. A regression model was also used to determine factors affecting ISQ measurements over time.

**Results:**

Treatment of peri‐implantitis resulted in significant improvements of both average PPDs and BOP (*p* < .0001 and *p* < .01). ISQ values marginally improved initially for all treatment modalities, but improvement was only maintained for 2–3 years in treatment modalities I (+1.28), III (+1.49), and IV (+2.92). There was a statistically significant negative linear correlation between average PPD and the ISQ values recorded both at baseline (*r* = −.618, *p* < 0.0001) and at 2/3 years (*r* = −.604, *p* < 0.0001).

**Conclusion:**

Over the 2–3‐year follow‐up period, all four treatment modalities led to improved clinical and radiographic peri‐implant parameters but implant stability posttreatment, as indicated by the fact that the recorded ISQ scores remained stable. As a result, use of MRFA as an adjunct to the traditionally used periodontal and radiographic tools for the evaluation of postoperative implant stability following the treatment of peri‐implant disease cannot be recommended.

## 1 INTRODUCTION

Peri‐implant disease is a relatively common entity following the placement of dental implants and a variety of clinical treatment approaches have been used in its management (Chan et al., 2023; Monje et al., 2023). Given the previous variations in disease criteria used to define peri‐implantitis (PI) in the existing literature, it is perhaps unsurprising that there is no consensus among dental professionals regarding a preferred treatment protocol to treat peri‐implantitis and significant empiricism exists in the approaches selected (Renvert & Polyzois, 2018). It appears that while nonsurgical treatment is effective in treating peri‐implant mucositis, its effect on the management of peri‐implantitis is more questionable (Suárez‐López Del Amo et al., 2016). Many surgical approaches to PI have been advocated but the rate of treatment success may also be relatively poor (de Waal et al., 2016). Nevertheless, surgical treatment can offer long‐term effectiveness (Berglundh et al., 2018).

A systematic review on the surgical regenerative treatment of PI in human clinical studies concluded that this therapy appears to offer a predictable approach to management of PI, but there was insufficient evidence to support the superiority of regenerative versus nonregenerative surgical treatment (Daugela et al., 2016). While animal research has demonstrated that osseointegration is possible to achieve on a previously contaminated implant surface (Alhag et al., 2008; Kolonidis et al., 2003; Mohamed et al., 2010), human studies have offered limited evidence to support the possibility that this can be routinely achieved in clinical practice (Renvert & Polyzois, 2018).

The question as to whether treatment of PI can result in true re‐osseointegration has been controversial in the literature and is primarily restricted to animal studies in order to be able to carry out histological analysis. (Almohandes et al., 2019; Persson et al., 1999, 2001; Schwarz et al., 2006; Sennerby et al., 2005). In one human case series, peri‐implantitis lesions were treated surgically using porous titanium granules following decontamination of the implant surface. Following 12 months of healing, one implant and surrounding structures were excised from only one site and histological and micro CT analyses were carried out, which indicated that that re‐osseointegration is possible in the human model (Wohlfahrt et al., 2011).

A systematic review by Renvert et al. (2009) investigated the evidence for re‐osseointegration following PI treatment at contaminated implant surfaces. It revealed that access surgery with closed healing positively affected the rate of re‐osseointegration compared to nonsurgical therapy. Additionally, surgical therapy and the use of adjunctive regenerative materials resulted in variable levels of re‐osseointegration. The authors concluded that while re‐osseointegration is possible, the extent to which it can be achieved is influenced by different implant surface characteristics and that surface decontamination alone will not achieve a substantial level of re‐osseointegration. Furthermore, the literature failed to identify a predictable method of achieving re‐osseointegration in a previously contaminated site, with the various techniques yielding a wide range of re‐osseointegration from 1% to 84% (Renvert et al., 2009; Schwarz et al., 2006). The application of such studies to the human model is difficult due to obvious ethical restrictions; hence, it has not been studied to a comprehensive degree.

The application of growth factors like enamel matrix derivatives, concentrated growth factors, and platelet‐rich fibrin membranes (PRF) to the regeneration of periodontal and peri‐implant defects is a new regenerative approach and the goal is to enhance wound healing events. PRF products, in the form of Leukocyte‐ and Platelet‐rich Fiblin (L‐PRF) were extensively used in this study. Hamzacebi et al., (2015) conducted the first human study to compare the effectiveness of L‐PRF application and conventional flap surgery in the treatment of peri‐implant bone defects. Nineteen patients with 38 implants were diagnosed with peri‐implantitis, with diagnostic criteria including probing depths of ≥5 mm and radiographic bone loss of ≥2 mm with BOP. The subjects were randomly assigned to either receive open flap debridement with adjunctive L‐PRF membranes or open flap debridement alone. At the 6‐month follow up, regardless of the defect configuration, the L‐PRF group demonstrated significantly higher clinical attachment level gain and mean probing depth reductions than the control group. Furthermore, the amount of keratinized tissue for the L‐PRF group also increased significantly.

With limited human histological evidence, conclusions regarding the potential of surgical procedures to induce re‐osseointegration on the implant surface are difficult. At this moment in time, the clinician must rely on surrogate indicators of re‐osseointegration, such as radiological evidence of bone–implant contacts and increasing implant stability over time following treatment, using technologies such as Magnetic resonance frequency analysis (MRFA). Current available evidence regarding the use of MRFA in assessing peri‐implant disease treatment outcomes is relatively scarce. The relationship between ISQ values and changes in peri‐implant parameters following nonsurgical and surgical interventions, such as plaque levels, BOP, probing depths or radiographic bone‐level changes, and defect fill, remains largely unexplored.

The aim of the present study was to investigate the impact of peri‐implantitis treatment upon clinical parameters and implant stability quotient values in the medium term and to ascertain if MRFA can be used as a tool to demonstrate postoperative healing following treatment of peri‐implantitis.

## 2 MATERIAL AND METHODS

Study participants were recruited from among patients referred for treatment at a dedicated peri‐implantitis clinic at Dublin Dental University Hospital, Dublin, Ireland.

Following initial assessment, consecutive patients aged >18 years were enrolled for treatment of peri‐implantitis. This study protocol received approval from the relevant local research ethics committee (St James' Hospital/AMNCH Research Ethics committee, Dublin, Ireland Ref: 2017‐05 list 19 (2) and 2018‐04 Chairman's action (7). Treatment of these cases involved different clinical approaches, based on the variety of clinical presentations of disease among this cohort. The present reporting considered the checklist items as proposed in the STROBE statement (von Elm et al., 2014).

All participants demonstrated the following features:

(a) Presence of bleeding and/or suppuration on gentle probing, increased probing depth compared to previous examination, and presence of bone loss beyond what would be viewed as initial bone remodeling (Berglundh at al., 2018; Schwarz et al., 2018).
(b) In the absence of previous examination data: at least one functioning, nonmobile, osseointegrated implant with probing pocket depths ≥6 mm and the presence of bleeding and/or suppuration on gentle probing or evidence of progressive peri‐implant bone loss/bone loss of ≥3 mm detected radiographically (standard intraoral or panoramic) (Berglundh at al., 2018; Schwarz et al., 2018).
(c) Compliance with efforts at oral hygiene and willing to adhere to a peri‐implant maintenance schedule over a 12‐month period.

The following patients were excluded from participation in the investigation: (a) patients with an uncontrolled medical condition, (b) pregnant or lactating mothers, (c) patients with implants that had previously been subjected to nonsurgical/surgical treatment for peri‐implantitis, (d) patients with implants restored with prosthetic supra‐structures that could not be removed easily in order to facilitate multiple examinations/maintenance and treatment, and (e) current smokers.

Before surgical treatment, all participants underwent nonsurgical treatment to reduce signs of clinical inflammation. This consisted of mechanical debridement of the affected implants, as well as instruction on and motivation to use self‐performed oral hygiene methods. Where possible, modification of the involved restorations to facilitate access for appropriate oral hygiene aids was conducted in conjunction with treatment. No surgery had been performed before nonsurgical therapy and the patient had demonstrated evidence of satisfactory compliance with oral hygiene methods (≤30% full‐mouth plaque score). Re‐evaluation was performed at 6 weeks.

There were four treatment groups: (1) Nonsurgical therapy as a monotherapy. (2) Nonsurgical therapy, followed by the open‐flap debridement approach, (3) Nonsurgical therapy, followed by open flap debridement and application of L‐PRF only (plugs and/or membranes), and (4) Nonsurgical therapy, followed by open flap debridement and application of hard tissue regenerative materials with or without L‐PRF.

For the nonsurgical treatment, the implant surface was debrided with titanium implant curettes (Wingrove; Paradise Dental Technologies) and subsequently decontaminated chemically with 3% hydrogen peroxide applied for 1 min, before being washed with sterile saline solution for 1 min. The nonsurgical treatment was the same for all four groups.

The surgical approach used in each case, and the use of biomaterials (if any), was based on the clinical judgment of the surgeons involved. In patients in whom more than one implant was affected by peri‐implantitis, all sites in the involved arch were treated simultaneously and using the same treatment method.

All surgical procedures were performed under local anesthesia. Before surgery, the prosthetic superstructure was removed to facilitate surgical access to the affected implant sites. A crestal incision connected affected sites and was extended to allow sufficient access to the associated bone defects. A full‐thickness mucoperiosteal flap was elevated to expose the alveolar crest beyond the apical border of the peri‐implant bone defects and granulation tissue was removed. The implant surface was debrided with titanium implant curettes (Wingrove; Paradise Dental Technologies) and subsequently decontaminated chemically with 3% hydrogen peroxide applied for 1 min, before being washed with sterile saline solution for 1 min.

In sites where reconstruction was attempted, the lingual and buccal mucoperiosteal flaps were repositioned and stabilized using tension‐free closure with a combination of horizontal mattress and single interrupted sutures. The prosthetic superstructure was cleaned and repositioned at the end of the surgical procedure.

In all groups, patients were prescribed systemic antimicrobial therapy (Amoxicillin 500 mg + metronidazole 200 mg each three times daily for 5 days) and instructed to rinse with 0.2% chlorhexidine digluconate mouthrinse (Corsodyl; GlaxoSmithKline Consumer Healthcare) twice daily for 14 days. Each patient received personalized instructions in oral hygiene methods according to their individual needs and supra‐structure design.

Patients were recalled at 3, 6, 12, and 24–36 months after surgery for peri‐implant maintenance appointments; these consisted of evaluation of oral hygiene, re‐instruction of and motivation to follow oral hygiene techniques, and supra‐gingival instrumentation when required. During these appointments, all supra‐structures were removed and at 6, 12, and 24–36 months, clinical and implant stability measurements were taken (Table 1).

**Table 1:** Research timeline.

	Baseline	3 months	6 months	12 months	24/36 months
Clinical measurements	✓		✓	✓	✓
MRFA (Osstell^tm^)	✓		✓	✓	✓
Treatment	✓				
Radiographs	✓			✓	✓
Removal of prosthesis and OH instructions	✓	✓	✓	✓	✓

Clinical evaluation included measurement of MRFA using an Osstell® ISQ device (Stampgatan), probing pocket depths, measurements of mucosal recession, presence of bleeding on gentle probing, and presence of plaque. All parameters were recorded at six sites per implant. At the end, the width of keratinized tissue was recorded.

As all patients represented inter‐clinic referrals to the peri‐implantitis clinic, patients had undergone recent radiographic exposure—most commonly orthopantomograms—before their attendance at our clinic. Additional images in the form of intraoral periapical films (long cone technique) were requested where the radiographic angulation or diagnostic quality of the provided images was not sufficiently satisfactory for surgical planning. Care was taken to minimize additional radiographs, following the principle of keeping patient exposure *as low as reasonably achievable*. Postoperative radiographs were taken at the 12‐ and 24–36‐month recall to evaluate the outcomes of treatment and/or to assess for additional treatment needs. The distance from the implant platform to the most coronal aspect of the bone was measured as the defect depth. This was compared between the baseline 12‐month and 24–36‐month time points. All radiographs taken were digital images recorded within the patient management system of the Hospital. The radiographic software used (Romexis, Planmeca) offers an inbuilt, calibrated tool for making dimensional measurements. As the X‐rays used ware not standardized, marginal bone‐level changes were reported but not included in the regression analysis.

### 2.1 Statistical analysis

A unique tabulated data collection sheet was used to collect data for each implant. All data were then transferred to Microsoft Excel® spreadsheets and following cleaning of the data, input into SPSS® software (IBM®) for statistical analysis.

No sample size calculation was performed as there were no studies available with a similar design that could be used for the calculations.

All data components were statistically analyzed at implant level using SPSS® software. Statistical significance and mean values were determined for normally distributed data using one‐way analysis of variance at the 0.05 significance level, and a Tukey posttest was performed when *p* < .05. A Kruskal–Wallis *H* test was performed for nonparametric data at the 0.05 significance level and Dunn posttest was performed when *p* < .05.

Subgroup analysis was performed to determine the effects of the various treatments carried out in the study.

**Figure 1:**
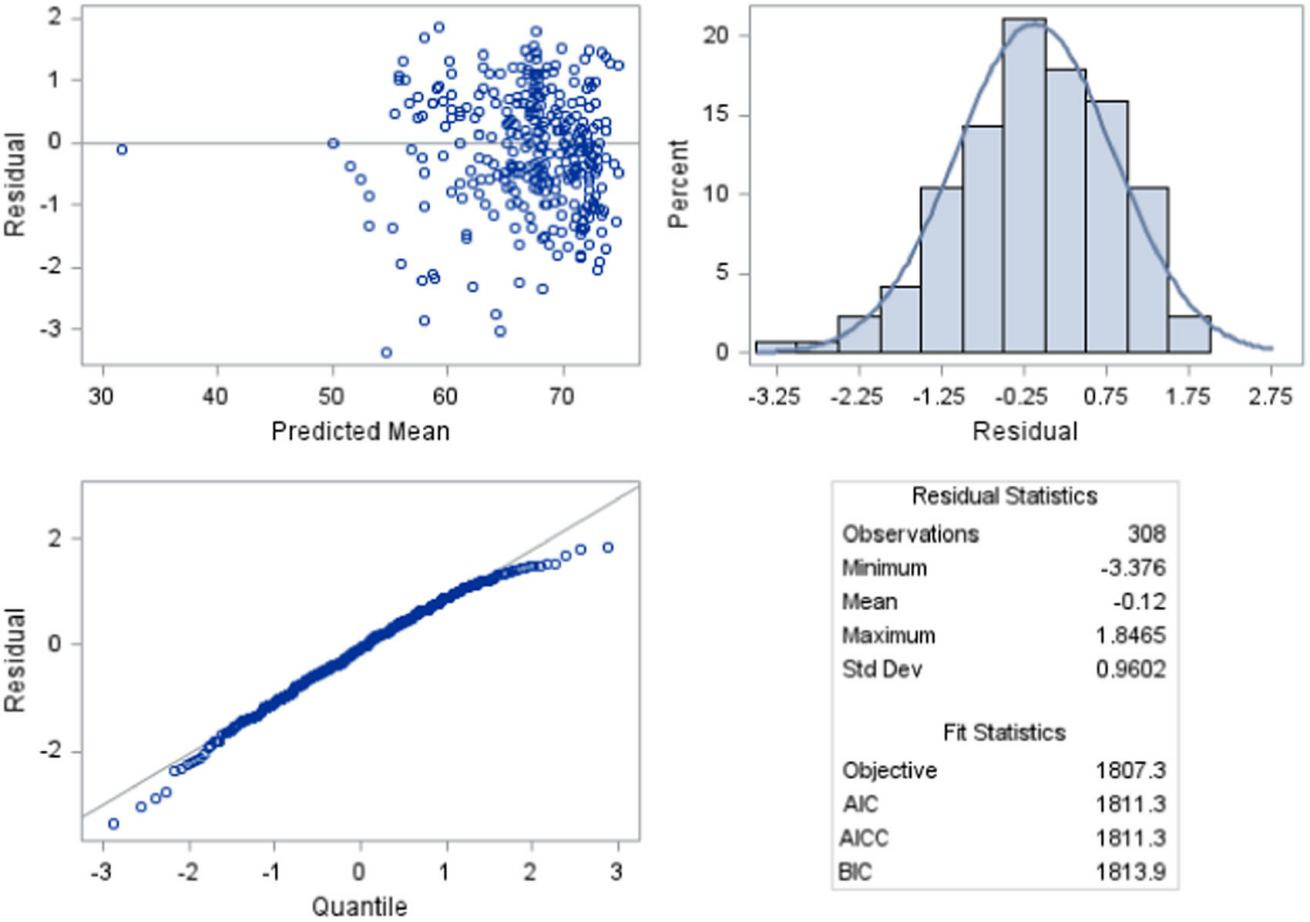
QQ plot and residual diagnostics for the regression model.

A regression model to determine factors affecting Osstell® (ISQ) measurements over time (dependent variable) with respect to four selected independent variables was developed using SAS v 9.4 PROC MIXED. The independent variables included in the model were treatment group (4), implant location (maxilla/mandible), average BOP over time, and average PPD over time. In addition to looking at the above‐named predictor variables, we also considered interactions with treatment group and implant location. All predictors and two‐factor interactions were included in the initial model. A manual backwards model selection method was then applied to arrive at the final, most optimal model. A QQ‐plot of the standardized residuals illustrated a linear pattern, thus indicating that residuals assumptions were satisfied (Figure 1).

Finally, in order to explore the nature of the relationship between ISQ measurement, average BOP, and average PPD, we looked at the linear correlation between ISQ measurements and both BOP and average PPD at all four time points.

## 3 RESULTS

Twenty‐six patients with 86 implants and ranging in age from 41 to 76 years agreed to participate in the study. Following nonsurgical treatment, three implants in three different patients were deemed hopeless and were removed. Finally, 25 patients (17 female and 8 male) with 83 implants were included in the study. The vast majority of implants were either Brånemark® (*n* = 31) (Nobel Biocare AB), Nobel Replace® (Nobel Biocare AB), or Biomet 3i® (*n* = 47) (Palm Beach Gardens). Additionally, 7 Ankylos® implants (Dentsply Sirona) and 1 Straumann® implant (Straumann Institute AG) were present. Out of all implants diagnosed with peri‐implantitis, 50 were supporting full‐arch fixed prostheses. Fourteen retained implant‐supported overdentures by means of a bar and seven by means of locator attachments. Three implants supported fixed partial dentures, while nine implants supported single unit implant restorations. Fourty‐five implants were located in the maxilla and 38 in the mandible.

**Figure 2:**
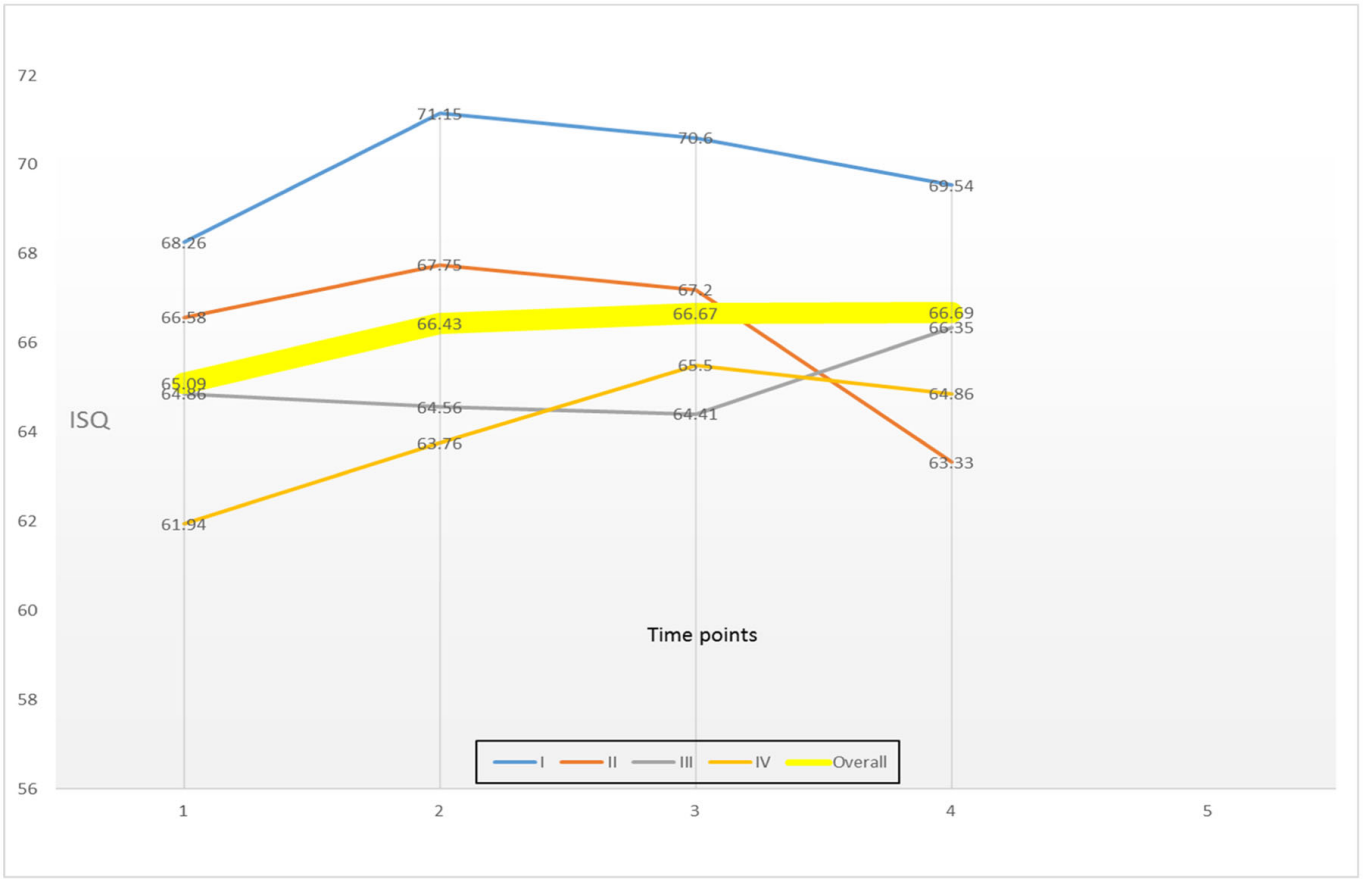
Mean ISQ values for all time points and for the four treatment modalities.

Following treatment, there was an overall mean radiographic bone‐level gain of 0.27 mm and a reduction in the mean implant plaque scores from 76.89% at baseline to 44.58% at 2/3 years. The deepest mean probing depth reduced by 1.55 mm, while the average probing depth reduced by 1.43 mm. There was an average clinical attachment gain of 1.25 mm and a reduction in mean bleeding on probing scores of 25.42%. Differences from baseline remained statistically significant across all three different time points (Table 2). There were no statistically significant changes in keratinized tissue levels. Finally, ISQ scores marginally increased from a mean baseline value of 65.09–66.69 at 2/3 years posttreatment; however, this was not statistically significant (*p* = .4684) (Table 2, Figure 2).

**Table 2:** Clinical and RFA outcomes (all modalities).

Variable	Baseline	6 months	12 months	24/36 months	
Marginal bone level (mm)	76		80	75	*N*
	3.97 (1.90)		3.53 (1.91)	3.70 (1.71)	Mean (SD)
					Sig. *p=*.311
Deepest probing depth (mm)	83	81	83	77	*N*
	5.84 (2.46)	3.85 (1.47)	3.92 (1.71)	4.29 (2.01)	Mean (SD)
					Sig. *p* < .0001
Average probing depth (mm)	83	81	83	77	*N*
	4.88 (2.30)	3.18 (1.29)	3.29 (1.62)	3.45 (1.76)	Mean (SD)
					Sig. *p* < .0001
Average clinical attachment loss (mm)	83	81	83	77	*N*
	5.38 (2.35)	3.73 (1.56)	3.92 (1.81)	4.13 (1.95)	Mean (SD)
					Sig. *p* < .0001
Bleeding on probing (%)	83	81	83	77	*N*
	57.02 (43.95)	30.04 (33.06)	31.72 (36.49)	31.60 (37.71)	Mean (SD)
					Sig. *p* < .01
Plaque score (%)	83	81	83	77	*N*
	76.89 (40.98)	41.97 (43.06)	49.99 (46.99)	44.58	Mean (SD)
					Sig. *p* < .0001
Keratinized tissue (mm)	83	81	81	77	*N*
	2 (1.91)	2 (1.64)	1.92 (1.70)	2.14 (1.73)	Mean (SD)
					Sig*. p* = .8368
ISQ score (1–100)	81	80	82	65	*N*
	65.09 (8.06)	66.43 (8.13)	66.67 (8.32)	66.69 (9.11)	Mean (SD)
					Sig*. p* = .4684

*Note*: Statistically significant differences from baseline in red.

Table 3 outlines the treatment subgroup analysis for all clinical and RFA outcomes at each time point. Table 4 reports the number of implants included in the analysis for each modality and at each time point. Nonsurgical therapy yielded statistically significant reductions in mean and deepest probing depths as well as gain in clinical attachment, which were largely maintained for 2–3 years.

**Table 3:** Treatment subgroup analysis for all clinical and RFA outcomes at each time point.

Variable	Modality	Baseline mean (SD)	6 months mean (SD)	12 months mean (SD)	24/36 months mean (SD)	Diff. baseline‐24/36	Sig.
Marginal bone level (mm)	I	3.17 (1.01)		2.68 (1.44)	2.70 (1.12)	+0.47 mm	*p* = .1177*
	II	2.79 (2.17)		3.03 (1.79)	3.85 (1.37)	−1.06 mm	*p* = .5041**
	III	4.46 (1.66)		4.28 (1.68)	4.48 (1.50)	−0.02 mm	*p* = .9460*
	IV	4.62 (2.19)		3.75 (2.24)	3.75 (2.03)	+0.87 mm	*p* = .2618**
Deepest probing depth (mm)	I	4.85 (1.42)	3.71 (0.95)	3.42 (1.02)	3.90 (2.14)	+0.95 mm	***p*** **< .0001** *
	II	4.41 (1.50)	2.75 (0.75)	3.08 (1.24)	3.44 (1.23)	+0.97 mm	***p*** **= .0302** *
	III	6.25 (2.42)	3.95 (1.71)	4.50 (2.20)	4.72 (2.25)	+1.53 mm	***p*** **= .049** *
	IV	6.92 (2.91)	4.48 (1.58)	4.11 (1.65)	4.56 (1.85)	+2.36 mm	***p*** **= .0001** *
Average probing depth (mm)	I	3.92 (1.28)	2.90 (0.84)	2.97 (0.88)	3.05 (1.73)	+0.87 mm	***p*** **= .0010** **
	II	3.48 (1.40)	2.38 (0.68)	2.51(1.05)	2.74 (1.08)	+0.74 mm	*p* = .0785**
	III	5.35 (2.16)	3.31(1.46)	3.83 (2.20)	3.97 (2.08)	+1.38 mm	***p*** **= .0052** *
	IV	5.87 (2.82)	3.69 (1.47)	3.40 (1.55)	3.58 (1.61)	+2.29 mm	***p*** **= .0001** *
Average clinical attachment level (mm)	I	4.06 (1.23)	2.97 (0.90)	3.12 (0.88)	3.19 (1.66)	+0.87 mm	***p*** **= .0161** *
	II	4.16 (1.84)	3.19 (1.53)	3.54 (1.86)	4.14 (1.83)	+0.02 mm	*p* = .4903**
	III	5.82 (2.03)	4.07 (1.76)	4.70 (2.16)	4.93 (1.91)	+0.89 mm	***p*** **= .0062** *
	IV	6.59 (2.78)	4.33 (1.56)	4.02 (1.78)	4.21 (2.036)	+2.38 mm	***p*** **= .0002** *
Bleeding on probing (%)		42% (45)	20% (26)	24% (33)	19% (29)	−23%	*p* = .4080*
	II	80% (22)	20% (21)	43% (43)	46% (46)	−34%	***p*** **= .0095** *
	III	47% (51)	40% (40)	35% (38)	37% (43)	−10%	*p* = .9399*
	IV	64% (39)	36% (35)	30% (35)	28% (35)	−36%	***p*** **= .0076** *
Plaque score (%)	I	48% (27)	18% (27)	52% (51)	29% (40)	−19%	*p* = .1296*
	II	88% (29)	55% (47)	62% (43)	87% (33)	−1%	*p* = .1626*
	III	78% (39)	55% (41)	54% (45)	50% (48)	−28%	*p* = .1253*
	IV	92% (25)	43% (46)	38% (46)	36% (46)	−56%	***p*** **= .0001** *
Keratinized tissue (mm)	I	2.57 (1.72)	2.85 (1.62)	2.76 (1.72)	2.80 (1.75)	+0.23 mm	*p* = .9006*
	II	0.33 (0.44)	0.91 (1.08)	0.75 (1.05)	0,66 (1.11)	+0.33 mm	*p* = .7403*
	III	2.18 (1.89)	1.95 (1.77)	1.87 (1.72)	2.15 (1.84)	−0.03 mm	*p* = .9342*
	IV	2.16 (2.15)	1.86 (1.48)	1.83 (1.63)	2.12 (1.53)	−0.04 mm	*p* = .9158*
ISQ score (1–100)	I	68.26 (4.81)	71.15 (5.91)	70.60 (6.65)	69.54 (7.89)	+1.28	*p* = .4932**
	II	66.58 (7.44)	67.75 (4.94)	67.20 (7.02)	63.33 (4.27)	−3.25	*p* = .3482*
	III	64.86 (7.24)	64.56 (7.05)	64.41 (7.33)	66.35 (7.20)	+1.49	*p* = .8134**
	IV	61.94 (10.19)	63.76 (10.15)	65.50 (10.03)	64.86 (12.58)	+2.92	*p* = .3676*

*Note*: Statistically significant differences from baseline in red. Bold values are statistically significant.

* Kruskal–Wallis test completed for non‐normally distributed data at the 0.05 significance level + Dunn posttest when *p* < .05.

** One‐way analysis of variance test completed for normally distributed data at 0.05 significance level + Tukey posttest when *p* < .05.

**Table 4:** Number of implants included in the analysis for each modality and at each time point.

Variable	Modality	Baseline *N*	6 months *N*	12 months *N*	24/36 months *N*
Marginal bone level	I	20		20	19
	II	09		12	07
	III	22		23	23
	IV	25		25	26
Deepest probing depth, average probing depth, average clinical attachment level, bleeding on probing, plaque score	I	21	21	21	21
	II	12	12	12	09
	III	24	23	24	22
	IV	26	25	26	25
Keratinized tissue	I	21	21	21	21
	II	12	12	12	09
	III	24	23	24	22
	IV	26	25	24	25
ISQ score (1–100)	I	21	20	20	21
	II	12	12	12	06
	III	23	23	24	20
	IV	25	25	26	18

An open flap approach, which encompassed surface decontamination and open flap debridement, resulted in a modest reduction of average probing depths (−0.74 mm), even though there was a very significant reduction in mean bleeding on probing levels.

The greatest reduction in deepest PPD and gain in CAL was observed in the IV group; a 2.29 mm average PPD reduction and a 2.38 mm gain in CAL at 2–3 years were found. In addition, there was also continued improvement in bleeding and plaque levels at all time points.

In tandem with changes in ISQ values, no statistically significant changes were detected. Overall, there was a minimal increase in the ISQ mean value at 6 months (+1.34). The implant stability was maintained at 12 and 24/36 months. The greatest increase in ISQ levels was observed at implants that received the regenerative treatment modality IV (+2.92), but this increase was not statistically significant (*p* = .4932) (Table 3, Figure 2).

For the regression analysis, all predictors selected (treatment groups, implant location, BOP, and PPD) and two‐factor interactions were included in the initial model. A manual backwards model selection method was then applied to arrive at the final, most optimal model. Based on the Type 3 tests shown in Table 5, both Visit and mean PPD had a statistically significant effect on ISQ measurements (*p* < .05). The (treatment group‐visit), (treatment group‐ average PPD), and (visit‐implant location) all also had a statistically significant effect on ISQ measurements (Table 5).

**Table 5:** Type 3 tests of fixed effects.

Effect	Num DF	Den DF	*F* Value	Pr > *F*
Visit	3	71	4.33	0.0074
Location (max/mand)	1	26	0.51	0.4829
Treatment modality	3	10	0.37	0.7763
Average PPD	1	258	75.13	<0.0001
Visit—Location	3	71	4.38	0.0069
Visit—Tx modality	9	26	3.08	0.0119
Av PPD—Tx modality	3	258	3.55	0.0151

Results from Pearson's correlation between ISQ measurements and PPD indicate a strong negative linear relationship between these two variables at all time points. As ISQ measurements increase, PPD linearly decreases (Table 6). Although significant, the correlation is weak between ISQ measurements and BOP.

**Table 6:** Pearson's correlation coefficients between ISQ measurement, BOP, and average PPD at all 4 time points.

	Baseline	6 months	12 months	2–3 years
Av PPD/ISQ	*r* = −.618	*r* = −.551	*r* = −.591	*r* = −.604
	*p* < .0001	*p* < .0001	*p* < .0001	*p* < .0001
BOP/ISQ	*r* = −.22	*r* = −.324	*r* = −.443	*r* = −.255
	*p* < .043	*p* < .0030	*p* < .0001	*p* < .0005

## 4 DISCUSSION

The aim of this study was to investigate the medium‐term impact of peri‐implantitis treatment upon clinical parameters and implant stability quotient values.

Overall, clinical and radiographic parameters improved over the study duration. There was a significant reduction in average and deepest probing depths at 6 months following treatment but a slight relapse was seen at 2/3 years. Similar observations were made for plaque scores and bleeding scores. Other studies have reported similar findings, that is, initial improvements following treatment and a degree of relapse in the subsequent follow‐up duration. Compliance with maintenance therapy and decreased standards of oral hygiene are often cited as critical factors (Heitz‐Mayfield et al., 2018; Lin et al., 2019; Monje et al., 2016). Rinke et al. (2011) demonstrated that patients who had received implants and did not comply with regular SPT visits had an 11‐fold increased risk of developing PI, and Ferreira et al. (2006) presented similar findings. However, one should keep in mind that these two studies refer to the importance of SPT in preventing the development of peri‐implantitis following the placement of implants and not of recurrence of peri‐implantitis following treatment.

Our results regarding nonsurgical therapy are in line with the findings from a recent systematic review by Cosgarea et al. (2022). On the contrary, results regarding PD reductions and gain in clinical attachment following the open flap approach were not in agreement with what has been reported in previous studies. Additionally, and in agreement with previous observations, relapse was common (Karlsson et al., 2022).

Enhanced clinical improvements were observed in the two regenerative treatment groups, which is line with the scientific literature to date. Some studies like the ones by Roccuzzo et al. (2011, 2017) have demonstrated greater improvements in clinical parameters following regenerative interventions. They treated single intrabony PI defects with a xenograft around TPS and SLA implants. The subjects were then followed up for 7 years. TPS surface implants showed a mean PPD reduction of 2.4 mm at twelve months and 3.8 mm at 7 years. SLA implants, meanwhile, showed a mean PPD reduction of 3.2 mm at 12 months and 3.4 mm at 7 years. In addition, there was a 35% reduction in BOP at 12 months and 60% at 7 years for the TPS group and 62.5% at 12 months and 67.5 at 7 years for the SLA group (Roccuzzo et al., 2011, 2017). Overall, available evidence is limited as there is considerable heterogeneity in the existing literature in terms of inconsistent inclusion criteria and types of biomaterials used.

When considering the application of L‐PRF membranes to the treatment of PI defects, Hamzacebi et al. (2015) demonstrated a mean PPD reduction of 2.82 mm for implants treated with open flap debridement and an adjunctive L‐PRF membrane, compared to a 2.05 mm decrease in the control group, which received access flap alone, at 6 months. This is consistent with changes in our study, where the incorporation of the L‐PRF membrane into the defect yielded a reduction in average PPD of 2.04 mm at 6 months, compared to a mean 1.1 mm reduction for the open flap debridement group.

No statistically significant changes in bone levels from baseline to 12 months were revealed in any of the treatment groups. When applied to the clinical scenario in everyday practice, a gain of 1 mm in peri‐implant bone levels following regenerative surgery would usually be clinically significant, particularly when combined with significant reductions in probing depths and inflammation (Herrera et al., 2023).

There were no significant changes in keratinized tissue levels over the 2/3‐year follow‐up period in any of the four treatment modalities, despite the inclusion of the L‐PRF membrane in treatment modality III or hard tissue grafting combined with L‐PRF in treatment modality IV. Following conventional treatment modalities, a gain in keratinized mucosa width would not usually be anticipated. However, the use of L‐PRF in specific cases alters such expectations. While the systematic review by Castro et al. (2017) focused on the gain in keratinized mucosa width around teeth, a mean difference of only 0.3 mm was found between the L‐PRF and connective tissue grafting groups, when combined with a coronally advanced flap at 6 months. If these findings were applied to the implants in our study, which received an L‐PRF membrane, one may have predicted greater gain in keratinized mucosa width for this cohort.

None of the four treatment modalities resulted in statistically significant changes in ISQ levels at any time point. However, for the nonsurgical group and the two regenerative groups, there was a modest overall increase in ISQ levels from baseline to the end of the observation period. The open flap group was the only group that demonstrated a mild decrease in stability 2–3 years following treatment. An interesting observation about the regenerative treatment group III, in which L‐PRF products were used as the sole grafting material, was that the pattern of the implant stability progression was very different to the other groups and the implants demonstrated increasing stability between 12 months and 2/3 years (Figure 2). It has been demonstrated that platelet concentrates and fibrin exudates are rich in growth factors and serum proteins promoting cell adhesion and migration into the fibrin clot. They also have mild antibacterial properties. These effects seem to have a positive impact on osseointegration, so one could suggest that they might have a similar effect on re‐osseointegration following peri‐implantitis treatment (Öncü et al., 2016, Schuldt et al., 2021).

In order to determine the true applicability of these results to clinical implant practice, the study limitations must be considered. Despite a total of 85 implants being treated, the most significant limitation in the study is the small number. This limitation is further emphasized when considering that the 85 implants were spread across 4 treatment groups. Implant level analysis must be mentioned as another limitation of the study herein. Finally, marginal bone‐level changes should be interpreted with caution as X‐rays used were not standardized. As a result, possible correlations between bone‐level changes and ISQ values could not be explored. Finally, the Osstell® transducers were tightened by hand and not with a torque‐controlled device and this might have affected the reliability of the ISQ measurements (Naughton et al., 2023).

## 5 CONCLUSIONS

Over the 2–3‐year follow‐up period, all four treatment modalities resulted in improved clinical and radiographic peri‐implant parameters but implant stability posttreatment, as indicated by the fact that the recorded ISQ scores remained stable. As a result, using MRFA as an adjunct to the traditionally used periodontal and radiographic tools for the evaluation of postoperative implant stability following the treatment of peri‐implant disease cannot be recommended.

## AUTHOR CONTRIBUTIONS

Edward Madeley contributed to the clinical treatment of patients and data collection. Michael Nolan contributed to the clinical treatment of patients and data collection. Peter Harrison contributed to the clinical treatment of patients. He conducted data analysis and interpretation. Stefan Renvert contributed to the conception and design of the study, and critically revised the manuscript. Ioannis Polyzois contributed to the conception and design of the study, contributed to the clinical treatment of patients, conducted data analysis and interpretation, and critically revised the manuscript. Peter Harrison, Edward Madeley, and Michael Nolan contributed equally to the study.

## CONFLICT OF INTEREST STATEMENT

Prof Renvert receives research grants and fees from Geistlich Pharma outside the present work. The remaining authors declare no conflict of interest.

## Data Availability

Data are available from the corresponding author upon reasonable request.
